# Selenium: a brief review and a case report of selenium responsive cardiomyopathy

**DOI:** 10.1186/1471-2431-13-39

**Published:** 2013-03-25

**Authors:** Abdulrahman Al-Matary, Mushtaq Hussain, Jaffar Ali

**Affiliations:** 1 Department of Neonatology, King Fahad Medical City, Riyadh, Kingdom of Saudi Arabia; 2 Neonatal ICU, Mid Western Regional Maternity Hospital, Ennis Road, Limerick, Ireland; 3 Department of Obstetrics and Gynaecology, Faculty of Medicine, University of Malaya, Kuala Lumpur, Malaysia; 4 Stem Cell Unit, Department of Anatomy, College of Medicine, King Saud University, Riyadh, Saudi Arabia

**Keywords:** Dilated cardiomyopathy, Glutathione, Peroxidase, Keshan disease, Selenoproteins, Selenium deficiency, Micronutrients deficiency

## Abstract

**Background:**

The authors review the role of selenium and highlight possible low selenium levels in soil that may result in deficient states in Saudi Arabia.

**Case presentation:**

The authors report a case of selenium-responsive cardiomyopathy in a 15-month old Saudi Arabian boy. This case of selenium deficiency causing dilated cardiomyopathy is presented with failure to thrive, prolonged fever and respiratory distress. The investigations revealed selenium deficiency. Selenium supplementation along with anti-failure therapy [Furosimide, Captopril] was administered for 6 months. Following therapy the cardiac function, hair, skin and the general health of the patient improved significantly.

**Conclusion:**

The patient with dilated cardiomyopathy of unknown etiology, not responding to usual medication may be deficient in selenium. Serum selenium measurements should be included in the diagnostic work-up to ensure early detection and treatment of the disease. The selenium level in the Saudi population needs be determined. Vulnerable populations have to undergo regular selenium measurements and supplementation if indicated. Dependence on processed foods suggests that the Saudi population fortify themselves with nutrient and micronutrient supplements in accordance to the RDA.

## Background

Selenium is an essential trace element in humans and animals. It is known for its potent antioxidant activity. Selenium is vital for good health [1-5]. Al-Saleh and coworkers [6] measured selenium in 300 umbilical cord blood samples collected from healthy pregnant women in Al-Kharj area in Saudi Arabia which showed serum selenium levels of 40.847 ± 8.969 μg/L in 300 newborns. Of interest, the cord serum selenium level was significantly lower in preterm infants than full-term infants (32 ± 8.029 μg/L versus 41.323 ± 8.784 μg/L). They also noted a significant positive correlation between selenium levels and birth weight. Their findings suggest that low selenium levels in newborns reflect an inadequate maternal dietary intake. Another study performed on Saudi Arabian patients suffering from dilated cardiomyopathy showed that while no link could be established between plasma selenium levels and the disease, the level of selenium in the supposedly healthy controls was at the lower end of the normal range and the test group only marginally lower than the control group [7]. In two other studies performed in the AlKharj area of Saudi Arabia by Al-Saleh et al. [8,9] noted that 41% of 737 adults [8] and 53.4% of 513 children [9] had toenail selenium levels lower than 0.56 μg/g that are considered low but not deficient (< 0.46 μg/g). Much earlier, studies performed by the same authors [10,11] on selenium levels in wheat grown in various parts of Saudi Arabia and*,* the soil, alfalfa and water in farms in AlKharj area of Saudi Arabia showed selenium levels to be insufficient in wheat grains of 7 of 8 regions in Saudi Arabia and very low in soil specimens. Importantly, the very low levels of selenium were found in the soil in the Keshan region of China, where selenium deficiency was thought to be the etiologic factor responsible for endemic dilated cardiomyopathy or Keshan disease [12,13] prevalent in that region. Indeed selenium deficiency is now well recognized as the causative factor implicated in the etiology of Keshan disease [14] in China.

This suggests that the Saudi population in the AlKharj region and possibly in the rest of Saudi Arabia could be at risk of selenium deficiency. The toenail selenium levels seen in Saudi Arabia were reflective of low but not deficient levels probably because unlike the Chinese of Keshan province the Saudis consume imported foodstuff, practice different lifestyles and their higher socioeconomic conditions probably help maintain their selenium levels within the normal range albeit at its lower end. This plus the findings of insufficient selenium observed in wheat grains grown in 7 of 8 regions of Saudi Arabia suggest that marginal selenium deficiency could be endemic in Saudi Arabia and that the deficient state could manifest in some isolated segments of the Saudi society. Selenium therefore deserves to be investigated in greater detail and such knowledge could assist in the diagnosis and resolution of disease conditions peculiar to selenium deficiency. Selenium is perhaps a hitherto neglected entity but possibly a crucial factor affecting the Saudi population. It is known to be essential for a vast number of normal physiologic activities and for the maintenance of good health. Therefore, its deficiency is implicated in an array of disease states which are discussed in this brief review.

Major dietary sources of selenium are plant foods (provided the soil is not deficient in selenium), animal kidneys, seafood, egg yolk and Brazil nuts [1,15,16]. Besides, the soil selenium level is reflected in the concentrations seen in plants [17]. Parenteral and enteral nutrition are iatrogenic causes of selenium deficiency. Selenium malabsorption, cystic fibrosis, rheumatoid arthritis, neoplasia, and other varied clinical disorders [18] exhibit low plasma levels of selenium.

Highest concentrations of selenium are found in Brazil nuts that provide 8 and 83 g Selenium/g [15]. Consumption of one and up to a maximum of two Brazil nuts per day (estimated to contain ~100 g of selenium) [15] may help to meet daily requirements. However, consumption of more than two Brazil nuts per day may contribute to selenium toxicity due to accumulation of selenium. In particular, high selenium exposure may be associated with defective glucose and lipid metabolism [19] and could lead to diabetes and hyperlipidemia.

Since the early 1900s selenium has been regarded as possessing anticancer properties [20]. It has been found to inhibit DNA damage *in vitro,* and reduce pulmonary metastasis and radiation-induced carcinogenesis *in vivo*[21-24]. There appears to be an association between selenium levels and risk of lung cancer [25]. Besides anticancer properties, selenium has a number of functions in the normal cardiovascular [26-28], reproductive [29-32], gastroenterological [33,34], hepatic [35] and immune systems [3,36,37]. In addition, new roles of selenium are being discovered [2,4,15,36,38-40].

Selenium in the form of various selenoproteins is involved in the human antioxidant systems [41]. The numbers of selenoproteins known to be operating in the human include at least 25 and up to 30 selenoproteins, including glutathione peroxidase, thioredoxin reductase, iodothyronine deiodinase, and selenoproteins P, W, and R [42,43]. It is now well documented that these enzymes protect against free radical damage. They also regulate DNA transcription and cell proliferation. Selenium exerts its chemopreventive effect through the glutathione and thioredoxin systems [41]*,* while some investigations suggest protective effects against allergies [44]*,* and asthma [45], the other studies could not find a correlation between selenium and asthma [46]. In addition, recent studies suggest growth inhibitory and proapoptotic activity for selenometabolites in premalignant cells [47]. It is fairly well recognized that selenium is also involved in thyroid function, T-cell immunity, and spermatogenesis [48]. It is also known to be a competitive antagonist of heavy metals such as arsenic and cadmium [49,50] that are potentially carcinogenic. Besides, selenium deficiency could predispose the affected individual to a number of diseases [51]. Selenium deficiency could predispose to viral infections or cause a depletion of selenium or both [3,37] as a consequence of which certain disease states could manifest such as cardiomyopathy (Coxsackie virus) or immunosuppression as in HIV-induced AIDS [52]. In particular, selenium deficiency in patients with HIV infection may be due to the trapping of selenium by a selenoprotein encoded by the proviral genome [53].

Numerous reports [14,18,51,54] have implicated selenium deficiency in cardiovascular diseases, including studies from Saudi Arabia [26,27], but other studies [39,55,56] including one study from Saudi Arabia [28] have been inconclusive. Congestive cardiomyopathy or skeletal*-* muscle disorders may also develop in patients with selenium deficiency [57].

Here the authors highlight the possible presence of low levels and possibly deficient states of selenium in the Saudi population. The authors report a case in point of selenium-responsive cardiomyopathy in a 15-month old Saudi Arabian boy. It is crucial to bear in mind that selenium deficiency could be the underlying cause when confronted with cardiomyopathy. This is due to the fact that selenium levels in the Saudi soil and therefore in its produce could be low. Indeed the level of selenium intake [26] and its level in the tested segments of the Saudi population appear to be low [7-9,26-28] as reviewed in this report. The early identification of selenium deficiency in dilated cardiomyopathy is imperative for the prevention and reversal of its deleterious effects on the myocardium [58,59].

## Case presentation

**Figure 1:**
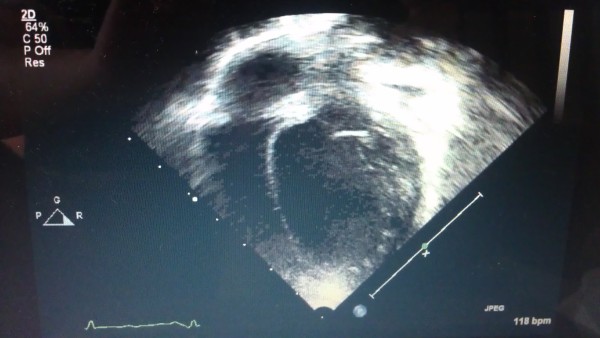
Echocardiography of a 15-month male infant with dilated myocardiopathy.

A 15 months old Saudi boy from the Al Qassem area of Saudi Arabia was presented with prolonged fever (6 months) and shortness of breath for two weeks. He had a history of chronic diarrhea and had lost 2 kg of weight in the previous 8 months. The patient presented with signs of congestive heart failure; labored breathing, tachypnea, poor appetite, weight loss, hepatomegaly and required anti-failure medications (Captopril and Furosemide). While these two medications helped to relieve the patient of his symptoms, they did not treat the underlying cause of the condition. Echocardiography (Figure 1) revealed dilated cardiomyopathy. He was born full term following normal spontaneous vaginal delivery. His birth weight was 3.4 kg. He was the 5^th^ sibling of non-consanguineous parents. His antenatal and post natal life periods were uneventful. There was no family history of any known chronic illnesses or unexplained death. He received breast-feeding exclusively for one year. He started sitting at 6 months of age and his development, weight and length were appropriate until six months prior to his presentation. Soon after six months of age he stopped to thrive due to ongoing illness. He had asthma which was controlled by ventolin and flixotide.

Investigations undertaken were his metabolic profile (serum ammonia, lactate, newborn screening, urine orgnic acid, tendem mass spectrophotometry (MS), serum carnitine level, organic acid gas chromatography-mass spectrophotometry (GC-MS), immunological profile [immunoglobulin level, oxidative burst test, lymphocytes markers], thyroid function tests, sweat chloride test, stool alpha 1 antitrypsin, celiac profile, bone profile, liver function test, coagulation profile, complete blood count, erythrocyte sedimentation rate (ESR), lipid profile, serum albumin, small intestinal biopsy, Magnetic Resonance imaging (MRI) brain. All were within normal limits, however his serum selenium level was very low [9.1 μg/l (measured by inductively coupled mass spectrophotometry); normal range: 55–103 g/l]. Interaction of selenium with other micronutrients was not investigated because thyroid function tests were normal and clinically there appeared to be no concern of iodine or vitamin E deficiency including that of other micro nutrients.

Selenium supplement (by the standard 2 μg/kg/day *which is well documented*) was commenced *per os* and was administered for about 6 months. His diarrhea, infections, failure to thrive, cardiomyopathy was managed in collaboration with the pediatric cardiology, gastroenterology, metabolic, neurology and nutritional units of the hospital. He was followed up in out-patient department.

**Figure 2:**
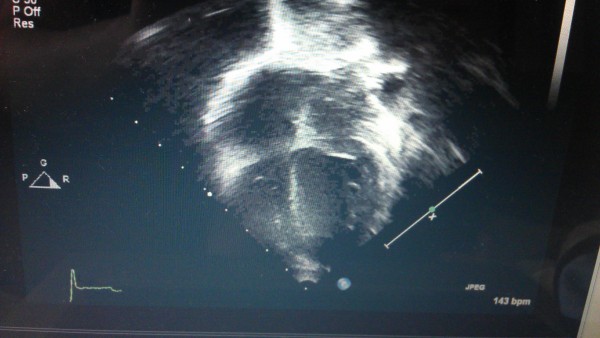
Apparently normal echocardiography after selenium therapy in a 15-month male infant.

Improvement was observed following these interventions. He gained weight. Positive changes were noted for his skin, hair and general health as evidenced by signs and symptoms and echo images. Repeat echocardiography was apparently normal following selenium treatment (Figure 2). He also demonstrated improved appetite, neurological and cardiac functions (ejection fraction was from 40 to 80%). The patient responded to selenium supplementation within 3–4 months. His selenium levels were repeatedly measured at 3 and 6 months following selenium supplementation that revealed normalized selenium levels (94/L).

Written informed consent was obtained from the patient’s parents for publication of this case report and any accompanying images. A copy of the written consent is available for review by the Editor of this journal.

## Conclusions

The prevalence of selenium deficiency and low levels of selenium in the Saudi population is not known. The available literature shows that highly plausible diseases associated with selenium deficiency appear to be a distinct possibility due to the low levels of selenium found in the soil, alfalfa grass, and water, and as reflected in the toenails in the area tested [6,8,9,11]. Although the soil selenium levels could vary from place to place, the very fact that low soil selenium exists in a given sector of Saudi Arabia suggests that it could likewise exist in other parts of the country. However the fact that the Saudi population depends almost entirely on imported processed foods suggests that the selenium deficiency could well be due to imported processed foods rather than from soil but this remains to be elucidated. Likewise it is probable that the dependence of the Saudi population on imported processed foods could also lead to deficiency in other nutrients and micronutrients. Clinicians must consider selenium deficiency if the symptoms and clinical presentations are reminiscent of diseases associated with selenium deficiency. The present case report is a case in point.

Although, the first cases were reported in 1937 during an epidemic in a rural area of China called Keshan, hence the term Keshan Disease [60], it was not until 1971 that a correlation between selenium deficiency and this disease was established. The mechanism of its pathogenesis is thought to be due to viral infection of selenium deficien*t*cy–induced weakened cardiomyocytes [61]. It is well documented that selenium is an essential co-factor of the enzyme glutathione peroxidase (GPX). There are 8 isoforms of GPX selenoenzymes in humans, but only six (GPX1-6) contain selenium. A decrease in the activity of glutathione peroxidase enzyme secondary to selenium deficiency may lead to an increase in the oxidative stress in the myocardial cells, and this can make the cell more susceptible to Coxsackie virus or infection by other viruses. This mechanism was demonstrated in mice models by injecting viral genome in mice deficient in selenium as opposed to mice fed diet containing sufficient selenium. The incidence of myocarditis in the former was much higher [62]. Furthermore, selenium deficiency appears to increase the virulence of otherwise benign virus. Myocardial necrosis, dyspnea, early fatigue and even early or sudden death are associated with selenium deficiency. The time course for the development of congestive cardiac failure in affected individuals varies in the presence of viral infection between several hours in patients with Keshan disease to several years in patients on total parenteral nutrition [63].

Selenium deficiency is confirmed by measuring serum or plasma concentrations of selenium. Values lower than 70 ng/mL or 0.8 μmol/L suggest that synthesis of selenium-associated proteins is not optimal and selenium supplies are limited [64]. Hovewer, selenium deficiency on its own does not usually cause illness. It makes the body more vulnerable to diseases [65].

Selenium and or glutathione peroxidase level(s) may be indicated if there is no other possible reason, cause or explanation for dilated cardiomyopathy in children. Selenium supplementation is indicated when it is below the normal range but should be discontinued when selenium levels are normalized. It may be worthwhile to determine the levels of selenium and other nutrients and micronutrients in the Saudi population due to the observation in a select Saudi community that selenium levels are on the border-line in the supposedly normal population (7). Moreover, it is recommended that regular selenium measurements are performed followed by supplementation if levels are below the normal range in selected vulnerable populations.

The dependence on imported processed foods in Saudi Arabia suggests that it may also be worthwhile to determine the levels of other nutrients including micronutrients in the Saudi population and common foods, since possible deficiencies of other nutrients and micronutrients could coexist with selenium deficiency. Until sufficient data is available to prove otherwise, the Saudi population should fortify themselves with nutrients and micronutrient supplements in keeping with the Recommended of Dietary Allowances (RDA) because processed foods may be deficient in nutrients and micronutrients.

In view of possible prevalence of low or deficient levels of selenium in the Saudi population it is imperative that selenium deficiency is considered when symptoms presentation is reminiscent of diseases associated with selenium deficiency. It is important the selenium level in the Saudi population is determined. Vulnerable populations have to be carefully monitored with regular selenium measurements and supplementation if indicated. Dependence on processed foods suggests that the Saudi population fortifies themselves with nutrient and micronutrient supplements in accordance to the RDA.

## Abbreviations

ESR: Erythrocyte sedimentation rate; GC-MS: Gas Chromatography - Mass Spectrophotometry; GPX: Glutathione peroxidase; MRI: Magnetic resonance imaging; MS: Mass spectrophotometry; RDA: Recommended of Dietary Allowances.

## Competing interests

The authors have no conflicts of interest whatsoever with regard to funding, commercial interests, etc. Authors declare no competing interests. No funding was sought for this work. No funding was received from any source other than routine operational budget that was not handled by the authors.

## Authors’ contributions

ARM contributed to the conceptualization, conduct of the study and wrote the first draft of the case report [40%] MH provided the clinical aspects of the study [30%] while JA reviewed the subject and re-wrote the manuscript [30%]. All authors read and approved the final manuscript.

## Pre-publication history

The pre-publication history for this paper can be accessed here:

http://www.biomedcentral.com/1471-2431/13/39/prepub
